# MiR-155 and its functional variant rs767649 contribute to the susceptibility and survival of hepatocellular carcinoma

**DOI:** 10.18632/oncotarget.11206

**Published:** 2016-08-11

**Authors:** Jiansong Ji, Min Xu, Jianfei Tu, Zhongwei Zhao, Jun Gao, Minjiang Chen, Jingjing Song, Haidong Zhu, Xingyao Cheng, Junguo Hui, Xilin Lan, Xiaoming Yang

**Affiliations:** ^1^ Department of Radiology, Affiliated Lishui Hospital of Zhejiang University, The Fifth Affiliated Hospital of Wenzhou Medical University, The Central Hospital of Zhejiang, Zhejiang 323000, P. R. China; ^2^ Department of Hepatobiliary Surgery, Beijing Chao-Yang Hospital Affiliated with Capital Medical University, Beijing 100043, P. R. China; ^3^ Department of Radiology, Zhong-da Hospital, Medical School, Southeast University, Nanjing 210009, P. R. China; ^4^ Department of Radiology, Lab-Yang, University of Washington, Seattle, WA 98109, USA

**Keywords:** miRNA, microRNA-155, susceptibility

## Abstract

Hepatocellular carcinoma (HCC) ranks the fourth common cancer and the third common cause of cancer mortality among Chinese population. The development of hepatocellular carcinoma (HCC) were confirmed to be involved in complex interactions between environmental and genetic factors. MicroRNAs (miRNAs) have been found to play an important role in tumorigenesis and metastasis. Emerging evidence suggested that upregulation of miR-155, one of the best characterized miRNAs, could serve as a promising marker for the diagnosis and prognosis of many cancers, except for HCC. In current we tested the hypothesis that functional variant rs767649 located in the flanking region of miR-155 gene contributes to the development and survival of HCC. We identified that functional variant rs767649 in miR-155 regulation region was associated with risk and survival of HCC. The minor allele of rs767649 was significantly associated with an increased risk of HCC (OR=1.23, 95% CI=1.11-1.36, P = 7.97×10^-5^). The genotype TT of rs767649 was significantly associated with a 1.94 fold poor survival of HCC (HR=1.94, 95% CI=1.01-3.79), while 1.15 fold for genotype AT (HR=1.15, 95% CI=1.06-1.25). Results showed that miR-155 was highly overexpressed in HCC tissues, compared with the adjacent normal tissues (P<0.001). The allele T contributes to higher expression of miR-155 in both the HCC tissues and the adjacent non-tumor tissues (P< 0.01). Our findings suggested that miR-155 and its functional variant rs767649 might contribute to the increased risk and poor prognosis of HCC, highlighting the importance of miR-155 in the prevention and prognosis of HCC.

## INTRODUCTION

Hepatocellular carcinoma (HCC) is one of the most common cancers in the world. According to a report from the National Office for Cancer Prevention and Control in China, HCC is the fourth common cancer and the third common cause of cancer mortality among Chinese population [1]. It is estimated that there will be 0.466 million new HCC cases and 0.422 new deaths occurring in 2015 [1]. Although chronic infection with hepatitis B virus (HBV) or hepatitis C virus (HCV) has a major role in the development of the disease, the exact molecular mechanism of HCC still remains uncertain.

MicroRNAs (miRNAs), a class of 21–25 nucleotide (nt) single-stranded non-coding RNAs and post-transcriptionally regulate gene expression by base pairing to their target sequences, have been found to play an important role in tumorigenesis and metastasis [2–4]. MiRNAs were found to be involved in many important functions in development, cell differentiation, and regulation of cell cycle and apoptosis, and expression was abnormally regulated in cancer by a variety of mechanisms including amplification, deletion, mutation, and epigenetic silencing [5–8]. Identifying clinical biomarkers and molecular targets for HCC, like microRNAs, may contribute to improving the rate of early diagnosis and survival of patients with this lethal disease [9, 10]. This would allow the clinicians to detect an early stage of HCC, monitor tumor dynamics, and predict sensitivity to treatment and prognosis [5, 11]. Among them, MiR-155 was one of the best characterized miRNAs [9, 12]. Its expression level is significantly changed and is associated with susceptibility and prognosis of many cancers, including breast cancer, lung cancer, esophageal cancer, and papillary thyroid cancer [12–18]. Very recently, Xie et al [17] identified that a functional variant rs767649 in miR-155 regulation region contributed to lung cancer risk and survival. However, the intensive roles and mechanism of miR-155 in susceptibility and prognosis of HCC are poorly understood.

In current study, we hypothesize that functional variant rs767649 located in the flanking region of miR-155 gene contributes to the development and survival of HCC. Thus, we conducted this study which aims to identify an useful biomarker for screening HCC, monitoring and predicting poor prognosis.

## RESULTS

### Characteristics of the study population

Totally we recruited 1500 HCC patients and 1500 matched healthy controls in this study. The distribution of the studied population was presented in Table 1. Similar age, sex ratio, consumption of alcohol and smoking in two groups of the samples in two stages (All P value > 0.05), while HBV infection significantly contributed to susceptibility of HCC (P<0.01).

**Table 1:** Comparison of HCC patients and controls by selective characteristics

Variables	Stage 1			Stage 2		
	Cases (n=500)	Controls (n=500)	P value	Cases (n=1000)	Controls (n=1000)	P value
Age (years)	58.7±3.2	58.5±2.7	0.286	52.3±4.1	52.1±4.8	0.316
Gender (male)	300 (60.0%)	310 (62.0%)	0.517	620 (62.0%)	612 (61.2%)	0.713
HBV infection (HBsAg +)	110 (22.0%)	51 (10.2%)	**P<0.01**	221 (22.1%)	84 (8.4%)	**P<0.01**
Smoking status						
Ever	100 (20.0%)	101 (20.2%)	0.937	192 (19.2%)	172 (17.2%)	0.246
Never	400 (80.0%)	399 (79.8%)		808 (80.8%)	828 (82.8%)	
Alcohol status						
Ever	120 (24.0%)	130 (26.0%)	0.465	199 (19.9%)	201 (20.1%)	0.911
Never	380 (86.0%)	370 (84.0%)		801 (80.1%)	799 (79.9%)	

### Functional variant rs767649 contributes to the development of HCC

First, we genotyped the functional variant rs767649 of miR-155, which has been identified to be associated with risk and survival of lung cancer previously. The genotype distributions of rs767649 between HCC cases and cancer-free controls in stage 1 are shown in Table 2. Logistic regression analysis revealed that the minor allele of rs767649 was significantly associated with an increased risk of HCC [additive model: adjusted Odds ratio (OR) = 1.25, 95% confidence interval (CI) = 1.04-1.49, P = 0.016] after adjusting for age, gender, HBV infection, smoking and drinking. Compared with the subjects with genotype AA, those with AT were more likely to infect HCC (OR=1.34, 95% CI=1.02-1.76), as well as those with TT (OR=1.48, 95% CI=1.03-2.15).

**Table 2:** Functional variant rs767649 and HCC risk in stage 1

Genotype	Cases	Controls	Adjusted OR (95% CI)^*^
AA	163 (32.6%)	200 (40.0%)	1.00 (reference)
AT	246 (49.2%)	225 (45.0%)	1.34 (1.02-1.76)
TT	91 (18.2%)	75 (15.0%)	1.48 (1.03-2.15)
AT+TT vs AA	337/163	300/200	1.37 (1.06-1.78)
TT vs AT+AA	91/409	75/425	1.26 (0.90-1.76)
T vs A			1.25 (1.04-1.49)
P trend			**0.016**

* Adjusted for age at diagnosis, gender, HBV infection, smoking status and alcohol status

Then, the results were replicated in an independent population (stage 2). As shown in Table 3, the association was also significant in stage 2. When merged together, T allele was significantly associated with HCC risk, compared with A allele (OR=1.23, 95% CI=1.11-1.36, P = 7.97×10^-5^). We also evaluated the association between rs767649 and HCC risk, stratified by HBV infection status. The results showed that HBV infection status didn’t changed the association materially (Table 4).

**Table 3:** Functional variant rs767649 and HCC risk in stage 2 and the total effect

Genotype	Stage 2			Total effect		
	Cases	Controls	Adjusted OR (95% CI)^*^	Cases	Controls	Adjusted OR (95% CI)^*^
AA	325 (32.5%)	382 (38.2%)	1.00 (reference)	488 (32.5%)	582 (38.8%)	1.00 (reference)
AT	489 (48.9%)	472 (47.2%)	1.22 (1.00-1.48)	735 (49.0%)	697 (46.5%)	1.26 (1.07-1.47)
TT	186 (18.6%)	146 (14.6%)	1.49 (1.15-1.94)	277 (18.5%)	221 (14.7%)	1.49 (1.21-1.85)
AT+TT vs AA	675/325	618/382	1.28 (1.07-1.54)	1012/488	918/582	1.31 (1.13-1.53)
TT vs AT+AA	186/814	146/854	1.33 (1.05-1.69)	277/1223	221/1279	1.31 (1.08-1.59)
T vs A			1.22 (1.07-1.39)			1.23 (1.11-1.36)
P trend			**1.79×10^-3^**			**7.97×10^-5^**

* Adjusted for age at diagnosis, gender, HBV infection, smoking status and alcohol status

**Table 4:** Stratified analyses of association between rs767649 and HCC risk

Genotype	Adjusted OR (95% CI)^*^	
	HBsAg +	HBsAg −
AA	1.00 (reference)	1.00 (reference)
AT	1.24 (0.99-1.55)	1.27 (1.06-1.52)
TT	1.47 (0.90-2.39)	1.50 (1.18-1.91)
T vs A	1.22 (1.01-1.66)	1.23 (1.09-1.39)
P trend	**0.035**	**8.92×10^-4^**

* Adjusted for age at diagnosis, gender, smoking status and alcohol status

### Functional variant rs767649 contributes to the survival of HCC

We also evaluated the relationship between rs767649 and HCC survival. In the follow-up period of 9 years, 1422 patients were kept and 1125 of them died. As shown in Table 5, we found rs767649 was significantly associated with poor HCC survival. The results of Cox regression analyses showed that the genotype TT of rs767649 was significantly associated with a 1.15 fold poor survival of HCC (HR=1.15, 95% CI=1.06-1.25), while 1.11 fold for genotype AT (HR=1.11, 95% CI=1.04-1.19).

**Table 5:** Functional variant rs767649 and HCC risk survival

Genotypes	Case	Overall survival	
		Events	HR (95% CI)^*^
AA	453	330	1.00 (reference)
AT	708	576	1.11 (1.04-1.19)
TT	261	219	1.15 (1.06-1.25)
AT+TT	969	795	1.13 (1.06-1.19)

* Adjusted for age at diagnosis, gender, HBV infection, smoking status and alcohol status

### Variant rs767649, expression level of miR-155 and HCC tissues

**Figure 1:**
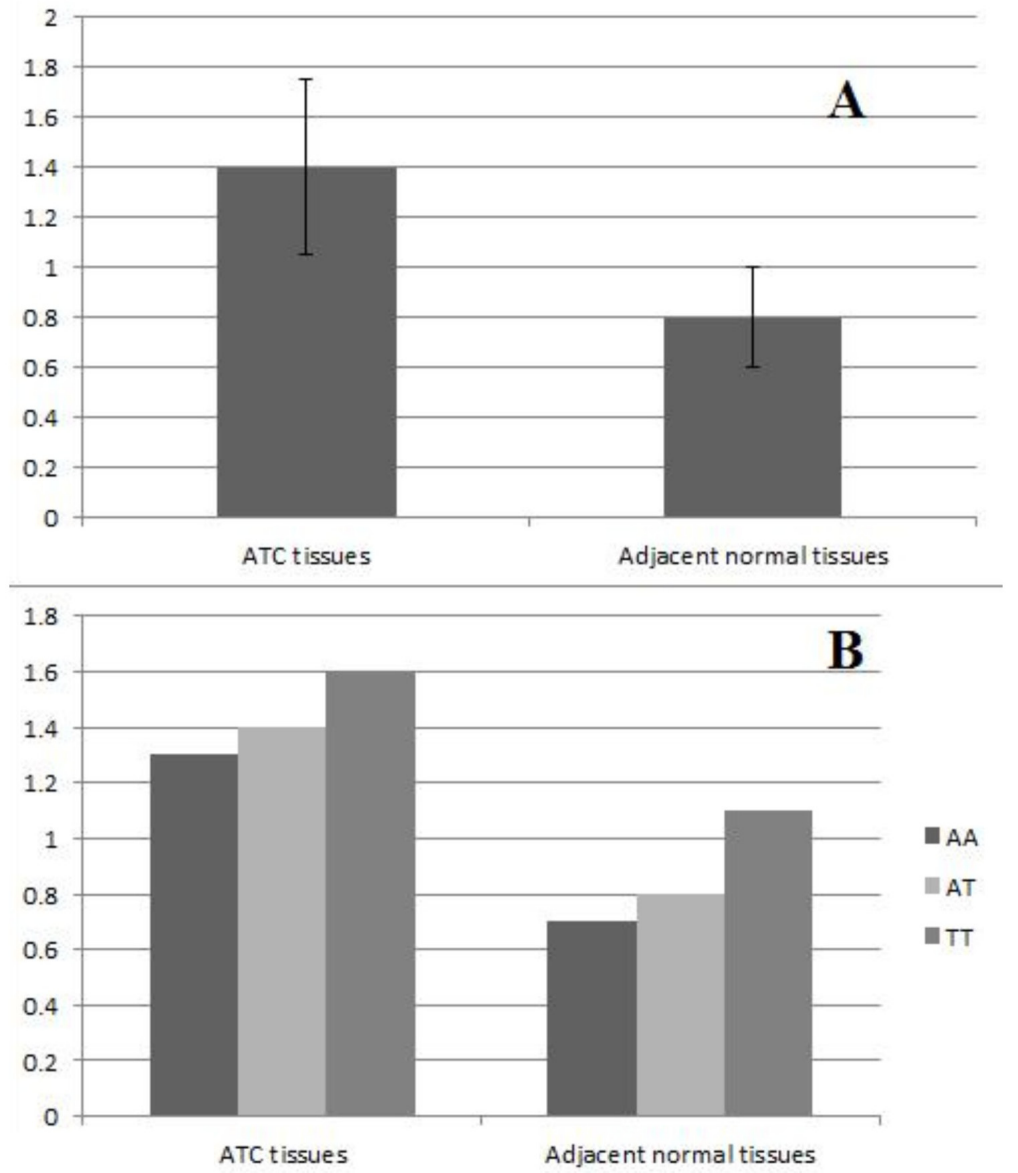
Comparison of miR-155 expression levels between HCC tissues and adjacent normal tissues **A.** Analysis showed that the relative expression levels of miR-155 in the HCC tissues were significantly higher than those in adjacent non-tumor tissues (P< 0.001). **B.** Variant rs767649 and expression level of miR-155 (P< 0.01).

To further explore the function of miR-155 in the development of HCC, we also tested the hypothesis that that expression level of miR-155 was associated with susceptibility of HCC in 200 randomly selected HCC patients. As shown in Figure 1A, the relative expression levels of miR-155 in the HCC tissues were significantly higher than those in adjacent non-tumor tissues (P< 0.001), which suggested that miR-155 may function as an oncogenic gene in the initiation of HCC. The allele T contributes to higher expression of miR-155 in both the HCC tissues and the adjacent non-tumor tissues (P< 0.01, Figure 1B), which is consistent with our previous hypothesis that T allele of rs767649 contributes to the higher risk of HCC.

## DISCUSSION

In this study, we investigated the association between miR-155 and its functional variant rs767649 and susceptibility and survival in the Chinese population using a two-stage case-control design. Consistent with our hypotheses, our findings suggested that miR-155 and its functional variant rs767649 which was located in the regulation region were associated with the increased risk and poor prognosis of HCC. To the best of our knowledge, this should be the first genetic association study to investigate miR-155 and its functional variant rs767649 on risk and survival of HCC.

HCC is one of the most aggressive tumors in human medicine. Previous studies revealed the necessity of genetic predisposing factors in the carcinogenesis of HCC [19–26]. Recently, microRNAs (miRNAs) has been revealed a pivotal role in cancer with increasing evidence showing that they may drive and potentiate oncogenesis [27–30]. Studies also found upregulation of miR-146b, miR-221, and miR-222, is observed in HCC and also in differentiated thyroid cancer, which indicated that these miRNAs’ overexpression was essential in maintaining tumorigenesis [31]. In current study, we found miR-155 was highly overexpressed in HCC tissues, compared with the adjacent normal tissues (P<0.001). These results were consistent with those in other related studies of cancers, including breast cancer, lung cancer, liver cancer, and so on [12, 17, 29, 32]. Consistent with Xie et al [17], we identified that functional variant rs767649 in miR-155 regulation region was also associated with risk and survival of HCC. MiR-155 was located at chr21:25573994-25574044, according to the UCSC genome browser, while SNP rs767649 was located at the intronic region of miR-155. According to HaploReg v4.1 [33, 34], a tool for exploring annotations of the noncoding genome at variants on haplotype blocks, such as candidate regulatory SNPs at disease-associated loci, we found that could change motifs, including Irf, Mrg1, Hoxa9, and PRDM1, which have been potential role in carcinogenesis.

Several limitations need to be addressed in our study. First, although we adopt the two-stage, case-control study design, which aims to make the results more reliable, the potential selection bias was Inevitable, and we still have limited statistical power to evaluated the gene-environment interaction relationship among the carcinogenesis of HCC. Second, due to the limited information about disease-free survival, we were unable to evaluate the role of rs767649 in disease-free survival of HCC. Strength this study should be the first and largest genetic association study to investigate candidate genes on risk and survival of HCC, the combination study of gene expression and functional variant which could in-depth study the essential role miR-155 in the tumorigenesis and development of HCC. Although further larger-scale studies are warranted to examine gene-gene and gene-environment interactions, this study had the acceptable statistical power to distinguish relatively small genotype association for rs767649.

In conclusion, this study provides strong evidence that miR-155 and its functional variant rs767649 may contribute to HCC susceptibility and prognosis, indicating biological significance of miR-155 in HCC carcinogenesis. However, larger, well designed studies with different populations and, in vitro and in vivo functional evaluations are warranted to confirm these findings.

## MATERIALS AND METHODS

### Patients and study samples

The study population has been described previously [35]. In brief, totally included in this study was samples from 1500 Chinese patients with sporadic HCC. The age and gender matched control group contained 1500 healthy Chinese population without any history of cancers, who were recruited from subjects who had annual physical examinations. Using a formatted questionnaire, which was used to collect demographic information, including age, sex, HBV infection status, smoking status, alcohol use, and family history of all cancer, face-to-face interviews were conducted among cases and healthy controls. Both approval from the appropriate institutional review board and written informed consents from patients who were included in this study were obtained. All patients were followed-up by personal or family contacts from the time of enrollment until death or the last scheduled of follow-up (July, 2014).

### DNA and RNA extraction, quantitative real-time PCR assay

Genomic DNA was extracted from 5 ml blood samples of all patients and controls using the GoldMag Whole Blood Genomic DNA Extraction kit in accordance with the manufacturer's instructions. Total RNA from the ovarian tissues were extracted with the Qiagen miRNeasy Mini kit (Qiagen), then was reversely transcribed to complementary DNA by using the TaqMan miRNA RT Kit (Applied Biosystems). MiRNA expression levels were tested using the TaqMan PCR kit, using SYBR PCR Master Mix reagent kits (TaKaRa). The results of miRNA expression were normalized using the threshold cycle (Ct) of β-actin.

### Genotyping

Genotyping was performed using SEQUENOM's MassARRAY® iPLEX assay (SEQUENOM Inc., San Diego, CA). The MassARRAY Typer 4.0 software was used for proper data acquisition and analysis. Genotypes were called after cluster analysis using the default setting of Gaussian mixture model. Inspection of the clusters was done to ensure a clear cluster separation with good signal to noise cutoff. A manual review was done to further clarify uncertain genotype calls. We selected 5% of the random samples for genotyping repeatedly, yielding a 100% concordance.

### Statistical analysis

SAS 9.3 (SAS Institute Inc., Cary, NC, USA) for Windows was used for statistical analysis. All p-values were two-tailed, and p<0.05 was considered statistically significant. Differences in the frequencies of the variants between the groups were analyzed using the two-tailed Fisher's exact test, the Student's t-test, or chi-square test. Odds ratios with 95% confidence intervals (CIs) were calculated for the risk estimate with logistic regression model. The joint effect of variables for survival of HCC was examined using the Cox Proportional Hazard regression model.
